# The ZJU index is a powerful surrogate marker for NAFLD in severely obese North American women

**DOI:** 10.1371/journal.pone.0224942

**Published:** 2019-11-26

**Authors:** Chia-Po Fu, Hira Ali, Vikrant P. Rachakonda, Elizabeth A. Oczypok, James P. DeLany, Erin E. Kershaw

**Affiliations:** 1 Division of Endocrinology and Metabolism, Department of Medicine, Taichung Veterans General Hospital, Taichung, Taiwan; 2 Department of Medicine, Chung Shan Medical University, Taichung, Taiwan; 3 Graduate Institute of Biomedical Electronics and Bioinformatics, College of Electrical Engineering and Computer Science, National Taiwan University, Taipei, Taiwan; 4 Division of Endocrinology and Metabolism, Department of Medicine, University of Pittsburgh, Pittsburgh, Pennsylvania, United States of America; 5 Division of Gastroenterology, Hepatology, and Nutrition, Department of Medicine, University of Pittsburgh, Pittsburgh, Pennsylvania, United States of America; 6 Translational Research Institute for Metabolism and Diabetes, Florida Hospital, Orlando, Florida, United States of America; University of Maryland, UNITED STATES

## Abstract

**Introduction:**

Non-alcoholic fatty liver disease (NAFLD) is the most common chronic liver disease in the western world and is highly associated with multiple cardiometabolic complications. The Zhejiang University (ZJU) index was first developed to predict NAFLD in Chinese populations, where it was shown to have better predictive value than other currently used indices. The aims of the present study were to 1) determine the diagnostic accuracy of ZJU index in identifying NAFLD in a well-phenotyped cohort of obese middle-aged American women and 2) compare its performance with other non-invasive indices for NAFLD identification.

**Methods:**

To achieve this goal, we performed a retrospective analysis of a prospectively-collected cohort of participants enrolled in a weight loss trial for severe obesity (RENEW, clinicaltrials.gov identifier: NCT00712127). One hundred and seven women between the age of 30 and 55 with obesity class II (BMI 35–39.9 kg/m^2^) or class III (BMI ≥ 40 kg/m^2^) were recruited for analyses. Hepatic steatosis was measured using liver/spleen attenuation ratio (L/S ratio) from unenhanced abdominal computed tomography. Beside ZJU index, hepatic steatosis index (HSI), lipid accumulation production index (LAPI), and visceral adiposity index (VAI) were also determined and to compare their performance in predicting NAFLD.

**Results:**

Of 107 obese women in the study, 40 (37.4%) met imaging criteria for NAFLD using cut-off value of L/S ratio < 1.1. The ZJU index was positively correlated with HIS, LAPI, but not VAI. The area under the curve was highest for the ZJU index (AUC = 0.742, 95% CI:0.647–0.837), followed by HSI (AUC = 0.728, 95% CI:0.631–0.825), LAPI (AUC = 0.682, CI:0.583–0.781), and VAI (AUC = 0.621, 95% CI:0.518–0.725), respectively, using the Youden method.

**Conclusion:**

The ZJU index is a powerful surrogate marker for NAFLD in severely obese western females and its predictive value was better than that of other commonly used indices for predicting NAFLD. Our study is the first to suggest that the ZJU index could be a promising model for use in western as well as Chinese populations.

## Introduction

Non-alcoholic fatty liver disease (NAFLD) is characterized by excessive accumulation of fat in hepatocytes in the absence of other recognizable causes, including alcohol consumption, viral infections, medications, or hereditary conditions [1,2]. It has become the most common etiology of chronic liver disease worldwide [3], with median prevalence of 20% in the general population using different diagnostic modalities, and as high as 30% in the United States [4]. More importantly, NAFLD is a systemic disease associated increased risk of cardiovascular disease [5,6] and other micro- or marcrovascular complications [7]. NAFLD had been linked with pro-atherogenic lipoprotein profile independent of traditional risk factors for cardiovascular disease [8]. Thus, early diagnosis and treatment of NAFLD can be critical for prevention of cardiovascular disease and its complications, reducing overall health care costs.

Liver biopsy is the gold standard for diagnosis and staging of NAFLD [9] and in research settings, NAFLD severity can be assessed with standardized histologic scoring systems, including the Nonalcoholic Steatohepatitis Clinical Research Network (NASH-CRN) or the Kleiner Modification of the Brunt scoring system [10]. However, liver biopsy is invasive, with up to 1% risk of serious complications. Furthermore, heterogeneous distribution of fibrosis and steatohepatitis can lead to sampling errors with liver biopsy. Therefore, noninvasive clinical scoring systems have been developed for identification of NAFLD, including Steatotest [11], Fatty liver index (FLI) [12], lipid accumulation production index (LAPI) [13], hepatic steatosis index (HSI) [14], and visceral adiposity index (VAI) [15]. The ZJU index [16] was derived from Chinese populations for identification of NAFLD, and has demonstrated greater sensitivity and specificity than FLI, HSI, LAPI, and VAI in those populations [17]. The ZJU index has not been studied in western populations.

Therefore, the aims of this study were 1) to evaluate the diagnostic accuracy of ZJU index in identifying NAFLD in a well-phenotyped cohort of obese American women and 2) to compare its performance with other non-invasive indices for NAFLD identification (HIS, LAPI, and VAI).

## Methods

### Study subjects

This was a retrospective analysis of a prospectively-collected cohort of participants enrolled in a weight loss trial for severe obesity (RENEW, clinicaltrials.gov identifier: NCT00712127) [18]. One hundred and seven women between the age of 30 and 55 with obesity class II (BMI 35–39.9 kg/m^2^) and class III (BMI ≥ 40 kg/m^2^) were recruited from the community from February 2007 to March 2009 at University of Pittsburgh Medical Center by way of television advertisements and mass mailing. The ethics committee of the University of Pittsburgh approved the study, and all subjects provided written informed consent. Inclusion and exclusion criteria were previously described [18–20]; in particular, study subjects had no history of coronary artery disease, diabetes mellitus, or uncontrolled hypertension. Participants with elevated liver enzyme greater than 30% above the upper limit of normal laboratory test were also excluded from the study.

### Demographic and anthropometric evaluation

Patient race and ethanol use were self-reported. Anthropometrics assessment included height and weight to calculate body mass index (BMI). Waist circumference (WC) was measured to the nearest 1 mm at the midpoint between lowest rib and the superior border of the iliac crest using an inelastic measuring tape while the participants stood on both feet with their arms hangings freely and recorded at the end of expiration. Using the 2005 National Cholesterol Education Program-Adult Treatment Panel III (NCEP-ATP III) criteria, metabolic syndrome was diagnosed if three or more of the following five criteria were present: waist circumference >40 inches (male) or > 35 inches (female), serum triglycerides ≥150 mg/dL, high-density-lipoprotein cholesterol (HDL-C) <40 mg/dL (male) or <50 mg/dL (female), blood pressure ≥130/85 mmHg, or fasting glucose ≥100 mg/dL [21].

### Laboratory investigation

All study subjects underwent blood testing at the same time of day following a 12-hour fast. Blood samples were collected for measurement of glucose, insulin, lipid profile, aspartate aminotransferase (AST), alanine aminotransferase (ALT), creatinine, high sensitivity-c reactive protein (hs-CRP), interleukin 6 (IL-6), adiponectin, and leptin. Homeostatic model assessment-insulin resistance (HOMA-IR), as a surrogate measure of insulin resistance, was calcuated using the following formula: blood glucose concentration (mmol/L) x the blood insulin concentration (mU/L)/22.5 [22]. The various indices for NAFLD scoring were calculated as follows: BMI was in kg/m^2^; fasting blood glucose (FBG) was in mmol/L; WC was in cm, triglycerides (TG) and high-density lipoprotein-cholesterol (HDL-C) were in mmol/L.

ZJU index = BMI + FBG + TG + 3x ALT/AST (+2 if female) [16].

Female VAI = [WC/(36.58+1.89xBMI)]x(TG/0.81)x(1.52/HDL-C) [15].

HSI = 8xALT/AST+BMI (+2 if female) [14]

Female LAPI = (WC-58)xTG [13].

### Non-contrast abdominal computed tomography

Hepatic steatosis was measured using liver/spleen attenuation ratio (L/S ratio) from unenhanced abdominal computed tomography (CT; 9800 CT scanner; General Electric, Milwaukee, WI) as previously described [18,23]. Non-contrast CT is a widely used method to assess hepatic steatosis with a sensitivity and specificity of 82% and 100%, respectively [24], depending on the cut-off value of L/S ratio used. As an L/S ratio < 1.1 exhibits over 80% accuracy for identification of 30% or greater macrovesicular steatosis, NAFLD was defined using an L/S ratio of < 1.1. Quantification of cross-sectional abdominal subcutaneous adipose tissue (AbdSAT) area and abdominal visceral adipose tissue (AbdVAT) area was done with CT measurement at the L4/L5 intervertebral level, as previously described [18,23]. Mid-thigh cross-sectional muscle area, subcutaneous adipose tissue area, subfascial adipose tissue area were also measured by non-contrast CT [18,23].

### Statistical analysis

Continuous variables are expressed as mean±standard deviation (SD) and categorical data as number and percentages. Continuous variables were compared using student t-test and Chi-square test was used to compare categorical data. The correlation between NAFLD model values and CT imaging parameters was determined by bivariate Pearson’s coefficients. Receiver operative characteristic (ROC) analysis curve was performed using NAFLD indices to identify NAFLD in severely obese women. Results were considered statistically significant at two-tailed *P* values of <0.05. All data were analyzed using SPSS 22.0 statistical software package (SPSS, Armonk, NY).

## Results

Of 107 obese women in the study, 40 (37.4%) met imaging criteria for NAFLD using cut-off value of L/S ratio < 1.1. Women with NAFLD had higher BMI, WC, FBG, insulin and insulin resistance, as measured by HOMA-IR. ALT, but not AST, was higher in women with NAFLD. Of the NAFLD indices, HSI, LAPI and ZJU index, but not VAI were higher in NAFLD group (Table 1). The NAFLD group had higher visceral adipose tissue (AbdVAT) area (204±71 vs. 170±67, *P* = 0.014) and total adipose tissue (AdbTAT) area (975±170 vs. 867±188, *P* = 0.003). Abdominal subcutaneous adipose tissue (AbdSAT) area, mid-thigh subcutaneous adipose tissue (MidthighSCAT) area, mid-thigh subfascial adipose tissue (MidthighSFAT) area, mid-thigh total adipose tissue (MidthighTAT) area and mid-thigh muscle (midthighMU) area did not differ between groups (Table 2).

**Table 1:** Baseline characteristics of NAFLD and non-NAFLD severe obese women.

	NAFLD (N = 40)	non-NAFLD (N = 67)	*P* value
Age (y)	46±6	47±7	0.652
Caucasian Americans, n (%)	29 (72.5)	38 (56.7)	0.191
**BMI (kg/m2)**	**46.0±5.7**	**42.1±4.8**	**<0.001**
**Waist circumference (cm)**	**126.2±10.9**	**119.2±11.0**	**0.002**
Systolic blood pressure (mmHg)	136±10	135±16	0.667
Diastolic blood pressure (mmHg)	77±7	78±9	0.853
**Fasting blood glucose (mg/dL)**	**97±12**	**90±10**	**0.005**
**Fasting insulin (uU/L)**	**18.1±9.1**	**14.3±7.9**	**0.027**
**HOMA-IR**	**4.4±2.5**	**3.3±1.9**	**0.012**
Total cholesterol (mg/dL)	192±37	187±29	0.478
HDL-C (mg/dL)	47±8	50±12	0.093
Triglyceride (mg/dL)	134±59	114±60.0	0.092
AST (U/L)	25.3±7.0	24.1±4.8	0.35
**ALT (U/L)**	**31.6±8.7**	**27.4±7.1**	**0.009**
Creatinine (mg/dL)	0.78±0.13	0.83±0.14	0.078
Metabolic syndrome, n(%)	28 (70)	34 (50.7)	0.11
Interleukin 6 (pg/mL)	2.9±1.7	2.7±1.9	0.529
Hs-CRP (mg/L)	9.9±8.3	8.1±7.9	0.276
**Leptin (ng/mL)**	**63.1±22.6**	**53.3±22.7**	**0.034**
Adiponectin (pg/mL)	6386±4874	6603±4296	0.814
**HSI**	**58.3±6.4**	**53.4±5.1**	**<0.001**
**LAPI**	**101.7±41.9**	**79.0±41.8**	**0.008**
VAI	1.7±1.0	1.5±1.2	0.28
**ZJU index**	**58.7±5.8**	**53.9±4.8**	**<0.001**

NAFLD: non-alcoholic fatty liver disease; BMI: body mass index; HOMA-IR: homeostatic model assessment-insulin resistance; HDL-C: high-density lipoprotein-cholesterol; AST: aspartate aminotransferase; ALT: alanine aminotransferase; hs-CRP: high sensitivity-c reactive protein; HSI: hepatic steatosis index; LAPI: lipid accumulation production index; VAI: visceral adiposity index.

**Table 2:** Baseline anthropometric measures of NALFD and non-NAFLD in severely obese women.

	NAFLD (N = 40)	non-NAFLD (N = 67)	*P* value
AbdSAT area (cm^2^)	766±185	699±170	0.058
**AbdVAT area (cm**^**2**^**)**	**204±71**	**170±67**	**0.014**
**AbdTAT area (cm**^**2**^**)**	**975±170**	**867±188**	**0.003**
MidthighSCAT area (cm^2^)	229±81	213±66	0.267
MidthighSFAT area (cm^2^)	18.5±5.8	18.2±6.1	0.84
MidthighTAT area (cm^2^)	252±82	236±68	0.261
MidthighMU area (cm^2^)	136±21	129±19	0.061

NAFLD: non-alcoholic fatty liver disease; AbdSAT: abdominal subcutaneous adipose tissue; AbdVAT: abdominal visceral adipose tissue; AbdTAT: abdominal total adipose tissue; MidthighSCAT: mid-thigh subcutaneous adipose tissue; MidthighSFAT: mid-thigh subfascial adipose tissue; MidthighTAT: mid-thigh total adipose tissue; MidthighMU: mid-thigh muscle.

**Fig 1:**
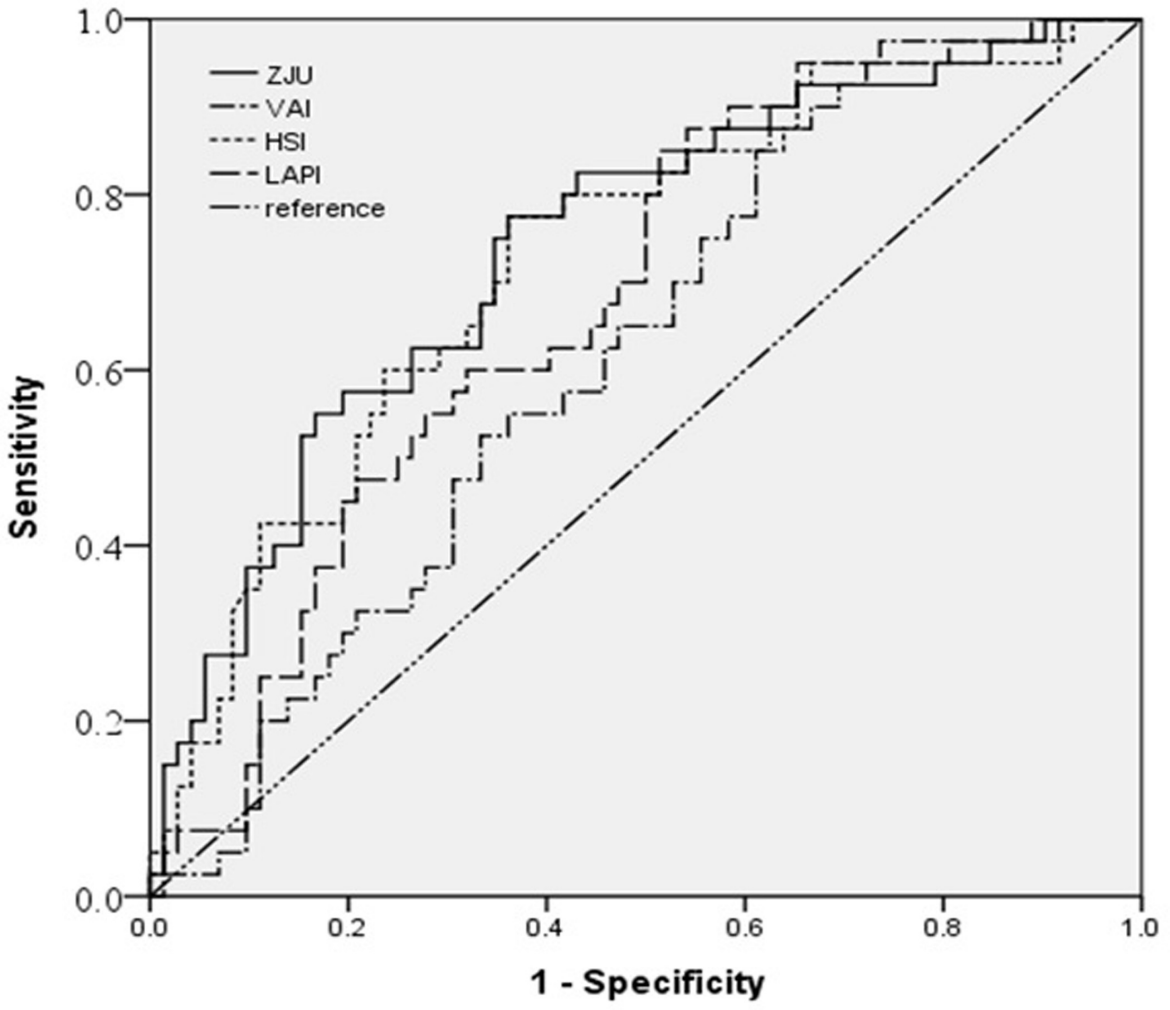
Receiver operative characteristic curve of ZJU index, HSI, LAPI and VAI for identifying NAFLD. VAI: visceral adiposity index; HSI: hepatic steatosis index; LAPI: lipid accumulation index.

Table 3 summarizes Pearson’s correlation coefficients; for NAFLD indices, L/S ratio and body fat distribution as measured by CT. L/S ratio was negatively associated with NAFLD indices (HSI, LAPI, VAI, ZJU index) and also with abdominal visceral adipose tissue area. The ZJU index was positively correlated with HIS, LAPI, but not VAI. In receiver operative characteristic analysis using NAFLD indices to identify NAFLD (L/S ratio <1.1), the area under the curve was highest in ZJU index (AUC = 0.742, 95% CI:0.647–0.837), followed by HSI (AUC = 0.728, 95% CI:0.631–0.825), LAPI (AUC = 0.682, CI:0.583–0.781), and VAI (AUC = 0.621, 95% CI:0.518–0.725) using Youden method, respectively (Fig 1, Table 4).

**Table 3:** Bivariate Pearson’s correlation coefficient between NAFLD indices and CT-mearsured regional fat.

	HSI	LAPI	VAI	ZJU index	L/S ratio	AbdSAT area	AdbVAT area	MidthighSCAT area
LAPI	0.166							
VAI	-0.051	0.859**						
ZJU	0.948**	0.286**	0.042					
L/S ratio	-0.243*	-0.361**	-0.228*	-0.275**				
AbdSAT area	0.670**	0.038	-0.189*	0.716**	-0.083			
AdbVAT area	0.316**	0.438**	0.343**	0.348**	-0.243*	0.068		
MidthighSCAT area	0.498**	-0.214*	-0.307*	0.523**	0.096	0.718**	-0.195*	
MidthighSFAT area	0.214*	0.037	-0.087	0.260**	-0.029	0.236*	0.181	0.101

*P<0.05,

**P<0.01.

NAFLD: non-alcoholic fatty liver disease; CT: computed tomography; HIS: hepatic steatosis index; LAPI: lipid accumulation production index; VAI: visceral adiposity index; L/S ratio: liver to spleen ratio; AbdSAT: abdominal subcutaneous adipose tissue; AbdVAT: abdominal visceral adipose tissue; MidthighSCAT: mid-thigh subcutaneous adipose tissue; MidthighSFAT: mid-thigh subfascial adipose tissue;

**Table 4:** Performance parameters of HSI, LAPI, VAI and ZJU for identifying NAFLD.

	AUC	95% CI of AUC	*P* value	Optimal cut off value	Sensitivity (%)	Specificity (%)
ZJU	0.742	0.647–0.837	<0.001	55.2	77.5	63.9
HSI	0.728	0.631–0.825	<0.001	54.41	77.5	63.9
LAPI	0.682	0.583–0.781	0.001	80.45	60	68.1
VAI	0.621	0.518–0.725	0.034	1.46	87.5	37.5

HSI: hepatic steatosis index; LAPI: lipid accumulation production index; VAI: visceral adiposity index; NAFLD: non-alcoholic fatty liver disease; AUC: area under the curve; CI: confidence interval.

## Discussion

The aim of our present study was to determine the performance of the ZJU index relative to other NAFLD prediction indices in a non-Chinese, western population, in this case North American severely obese women. The main findings of our study are that ZJU index could be a powerful surrogate indicator of NAFLD in severely obese North American women, and that it is better at identifying NAFLD than currently used indices including HSI, LAPI, and VAI.

Over the years, several indices have been developed in an effort to identify NAFLD using anthropometric and biochemical profile rather than invasive procedures such as liver biopsy [25]. However, no single index has been shown to be accurate in all populations and races. The ZJU index was first developed in 2015 specifically for identification of NAFLD in Chinese populations [16]. In our study, data were analyzed from a total of 107 women with class II and III obesity. We found that the ZJU index, HSI, and LAPI were significantly higher in the NAFLD group, but not VAI. The ZJU index and the HSI also take ALT/AST ratio into weighted consideration while LAPI only used WC and TG. VAI uses four parameters: BMI, WC, HDL-C, TG. Regarding the latter, neither HDL-C nor TG is significantly different in our subjects, and WC is partially adjusted by BMI according to the formula that these reasons may account for lack of significant difference in VAI between NAFLD and non-NAFLD groups in this study, and suggest that these variables may not be the most useful in a NAFLD prediction index. In contrast, stronger associations of NAFLD with the ZJU index and the HIS support the use of weighted ALT/AST in indices for identifying NAFLD.

In our study, the ZJU index performed better in identifying NAFLD than HSI, LAPI, VAI in severely obese North American women. The optimal cut off value for the ZJU index is > 55.2, which indicates that in severely obese women with BMI greater than 35 kg/m^2^ are more likely have NAFLD with AUC 0.742 and 77.5% sensitivity and 63.9% specificity. The other indices of AUC of NAFLD were 0.728, 0.682 and 0.621 for HSI, LAPI and VAI, respectively. These data provide evidence that the ZJU index may be a promising model in predicting NAFLD in the Western populations. When the ZJU index is > 55.2 in women with BMI greater than 35 kg/m^2^, individuals are more likely to have NAFLD and may benefit from additional radiological imaging for confirmation. The goal of a screening test in clinical practice is to achieve high sensitivity, and on that end, the VAI may actually be the best screening tests by given its high sensitivity of 77.5%. However, in our subjects with obese women, both sensitivity and specificity are pivotal given high prevalence of NALFD in such participants. Furthermore, in the era of value-driven health care, it is also beneficial to optimize specificity to minimize unnecessary imaging as well.

Cross-sectional abdominal visceral adipose tissue area is higher in NAFLD group, indicating that participants with NAFLD have more visceral fat. This finding is consistent with the negative association between abdominal visceral adipose tissue and L/S ratio (r = -0.243, *P* = 0.01). Previous studies have demonstrated that mid-thigh subfascial adipose tissue is associated with insulin resistance in patients with type 2 diabetes mellitus [26,27]. In our study, we did not find mid-thigh subfascial adipose tissue to be significantly associated with HOMA-IR (r = 0.073, *P* = 0.449), or L/S ratio (r = -0.029, *P* = 0.763), although significant with abdominal visceral adipose tissue was borderline (r = 0.181, *P* = 0.056). Rocha PM et al even reported that mid-thigh subfascial adipose tissue is positively associated with L/S ratio, suggesting a preventive role against ectopic hepatic steatosis in overweight and obese Caucasian women [28].

Our study showed lower AUC for ZJU index than Wang et al and Li et al [16,17]. There are several possible reasons for this result. First, the two study populations had several key differences. Our study subjects had class II and III obesity with an average BMI of approximately 43.5 kg/m^2^, whereas their study subjects were much leaner, with an average BMI of approximately 24 kg/m^2^. In addition, our study subjects were women, whereas their study subjects included both men and women. Second, to validate the ZJU index, the diagnosis of NAFLD was confirmed by liver biopsy in Wang et al [16] and by hepatic ultrasound in Li et al [17], whereas our study used L/S ratio by non-contrast CT. The latter is more objective than ultrasound, less costly and time-consuming than MR imaging. In addition, in Wang et al [16], liver biopsy was performed with unexplained abnormal liver function or during cholecystectomy with suspected NAFLD, whereas we excluded subjects with elevated liver enzyme greater than 30% above the upper limit of normal laboratory test. These differences may contribute to the relatively minor differences in performance of the ZJU in these different studies.

To our knowledge, the present study is the first study to evaluate on the ZJU index in a western population. Our study had some limitations. First, liver biopsies were not performed in our study subjects due to they are relative healthy besides obesity. Second, our study only includes obese women, thus the results may not be applicable to leaner populations or to men. Third, our sample size is relatively small, and may benefit expanding to a larger and/or broader population of western individuals.

## Conclusion

the ZJU index may be a powerful surrogate marker for NAFLD in severely obese western female. In our study, its diagnostic performance was better than that of other commonly used indices for identifying NAFLD such as HSI, VAI and LAPI. Our study is the first to suggest that the ZJU index could be a promising model for use in western populations. Further studies are needed to validate this finding in more diverse western populations.

## Supporting information

S1 File Baseline individual demographic data. (XLSX)

## References

[1] McCullough AJ. The clinical features, diagnosis and natural history of nonalcoholic fatty liver disease. Clin Liver Dis. 2004;8(3):521–33, viii. 10.1016/j.cld.2004.04.004 15331061

[2] Rinella ME. Nonalcoholic fatty liver disease: a systematic review. JAMA. 2015;313(22):2263–73. 10.1001/jama.2015.5370 26057287

[3] Sattar N, Forrest E, Preiss D. Non-alcoholic fatty liver disease. BMJ. 2014;349:g4596 10.1136/bmj.g4596 25239614 PMC4168663

[4] Vernon G, Baranova A, Younossi ZM. Systematic review: the epidemiology and natural history of non-alcoholic fatty liver disease and non-alcoholic steatohepatitis in adults. Aliment Pharmacol Ther. 2011;34(3):274–85. 10.1111/j.1365-2036.2011.04724.x 21623852

[5] Targher G, Byrne CD, Lonardo A, Zoppini G, Barbui C. Non-alcoholic fatty liver disease and risk of incident cardiovascular disease: A meta-analysis. J Hepatol. 2016;65(3):589–600. 10.1016/j.jhep.2016.05.013 27212244

[6] Ekstedt M, Hagstrom H, Nasr P, Fredrikson M, Stal P, Kechagias S, et al Fibrosis stage is the strongest predictor for disease-specific mortality in NAFLD after up to 33 years of follow-up. Hepatology. 2015;61(5):1547–54. 10.1002/hep.27368 25125077

[7] Targher G, Day CP, Bonora E. Risk of cardiovascular disease in patients with nonalcoholic fatty liver disease. N Engl J Med. 2010;363(14):1341–50. 10.1056/NEJMra0912063 20879883

[8] Siddiqui MS, Fuchs M, Idowu MO, Luketic VA, Boyett S, Sargeant C, et al Severity of nonalcoholic fatty liver disease and progression to cirrhosis are associated with atherogenic lipoprotein profile. Clin Gastroenterol Hepatol. 2015;13(5):1000–8 e3. 10.1016/j.cgh.2014.10.008 PMC4395517 25311381

[9] Chalasani N, Younossi Z, Lavine JE, Diehl AM, Brunt EM, Cusi K, et al The diagnosis and management of non-alcoholic fatty liver disease: practice Guideline by the American Association for the Study of Liver Diseases, American College of Gastroenterology, and the American Gastroenterological Association. Hepatology. 2012;55(6):2005–23. 10.1002/hep.25762 22488764

[10] Angulo P, Hui JM, Marchesini G, Bugianesi E, George J, Farrell GC, et al The NAFLD fibrosis score: a noninvasive system that identifies liver fibrosis in patients with NAFLD. Hepatology. 2007;45(4):846–54. 10.1002/hep.21496 17393509

[11] Poynard T, Ratziu V, Naveau S, Thabut D, Charlotte F, Messous D, et al The diagnostic value of biomarkers (SteatoTest) for the prediction of liver steatosis. Comp Hepatol. 2005;4:10 10.1186/1476-5926-4-10 16375767 PMC1327680

[12] Bedogni G, Bellentani S, Miglioli L, Masutti F, Passalacqua M, Castiglione A, et al The Fatty Liver Index: a simple and accurate predictor of hepatic steatosis in the general population. BMC Gastroenterol. 2006;6:33 10.1186/1471-230X-6-33 17081293 PMC1636651

[13] Bedogni G, Kahn HS, Bellentani S, Tiribelli C. A simple index of lipid overaccumulation is a good marker of liver steatosis. BMC Gastroenterol. 2010;10:98 10.1186/1471-230X-10-98 20738844 PMC2940930

[14] Lee JH, Kim D, Kim HJ, Lee CH, Yang JI, Kim W, et al Hepatic steatosis index: a simple screening tool reflecting nonalcoholic fatty liver disease. Dig Liver Dis. 2010;42(7):503–8. 10.1016/j.dld.2009.08.002 19766548

[15] Amato MC, Giordano C, Galia M, Criscimanna A, Vitabile S, Midiri M, et al Visceral Adiposity Index: a reliable indicator of visceral fat function associated with cardiometabolic risk. Diabetes Care. 2010;33(4):920–2. 10.2337/dc09-1825 20067971 PMC2845052

[16] Wang J, Xu C, Xun Y, Lu Z, Shi J, Yu C, et al ZJU index: a novel model for predicting nonalcoholic fatty liver disease in a Chinese population. Sci Rep. 2015;5:16494 10.1038/srep16494 26568423 PMC4645098

[17] Li L, You W, Ren W. The ZJU index is a powerful index for identifying NAFLD in the general Chinese population. Acta Diabetol. 2017;54(10):905–11. 10.1007/s00592-017-1024-8 28698957

[18] Goodpaster BH, Delany JP, Otto AD, Kuller L, Vockley J, South-Paul JE, et al Effects of diet and physical activity interventions on weight loss and cardiometabolic risk factors in severely obese adults: a randomized trial. JAMA. 2010;304(16):1795–802. 10.1001/jama.2010.1505 20935337 PMC3082279

[19] Rachakonda V, Wills R, DeLany JP, Kershaw EE, Behari J. Differential Impact of Weight Loss on Nonalcoholic Fatty Liver Resolution in a North American Cohort with Obesity. Obesity (Silver Spring). 2017;25(8):1360–8. 28605159 10.1002/oby.21890

[20] Rachakonda VP, Reeves VL, Aljammal J, Wills RC, Trybula JS, DeLany JP, et al Serum autotaxin is independently associated with hepatic steatosis in women with severe obesity. Obesity (Silver Spring). 2015;23(5):965–72. 25865747 10.1002/oby.20960 PMC4414671

[21] Grundy SM, Cleeman JI, Daniels SR, Donato KA, Eckel RH, Franklin BA, et al Diagnosis and management of the metabolic syndrome: an American Heart Association/National Heart, Lung, and Blood Institute Scientific Statement. Circulation. 2005;112(17):2735–52. 10.1161/CIRCULATIONAHA.105.169404 16157765

[22] Matthews DR, Hosker JP, Rudenski AS, Naylor BA, Treacher DF, Turner RC. Homeostasis model assessment: insulin resistance and beta-cell function from fasting plasma glucose and insulin concentrations in man. Diabetologia. 1985;28(7):412–9. 10.1007/bf00280883 3899825

[23] Goodpaster BH, Kelley DE, Wing RR, Meier A, Thaete FL. Effects of weight loss on regional fat distribution and insulin sensitivity in obesity. Diabetes. 1999;48(4):839–47. 10.2337/diabetes.48.4.839 10102702

[24] Park SH, Kim PN, Kim KW, Lee SW, Yoon SE, Park SW, et al Macrovesicular hepatic steatosis in living liver donors: use of CT for quantitative and qualitative assessment. Radiology. 2006;239(1):105–12. 10.1148/radiol.2391050361 16484355

[25] Machado MV, Cortez-Pinto H. Non-invasive diagnosis of non-alcoholic fatty liver disease. A critical appraisal. J Hepatol. 2013;58(5):1007–19. 10.1016/j.jhep.2012.11.021 23183525

[26] Kelley DE, McKolanis TM, Hegazi RA, Kuller LH, Kalhan SC. Fatty liver in type 2 diabetes mellitus: relation to regional adiposity, fatty acids, and insulin resistance. Am J Physiol Endocrinol Metab. 2003;285(4):E906–16. 10.1152/ajpendo.00117.2003 12959938

[27] Goodpaster BH, Thaete FL, Kelley DE. Thigh adipose tissue distribution is associated with insulin resistance in obesity and in type 2 diabetes mellitus. Am J Clin Nutr. 2000;71(4):885–92. 10.1093/ajcn/71.4.885 10731493

[28] Rocha PM, Barata JT, Minderico CS, Silva AM, Teixeira PJ, Sardinha LB. Visceral abdominal and subfascial femoral adipose tissue have opposite associations with liver fat in overweight and obese premenopausal caucasian women. J Lipids. 2011;2011:154672 10.1155/2011/154672 21961071 PMC3179871

